# Serum IgE Levels Are Associated With the Prognosis of Minimal Change Disease

**DOI:** 10.3389/fimmu.2022.840857

**Published:** 2022-03-17

**Authors:** Heng Li, Lefeng Wang, Xiayu Li, Wenqing Chen, Ying Zhang, Jianghua Chen

**Affiliations:** ^1^ Kidney Disease Center, First Affiliated Hospital, College of Medicine, Zhejiang University, Hangzhou, China; ^2^ Key Laboratory of Kidney Disease Prevention and Control Technology, Hangzhou, China; ^3^ National Key Clinical Department of Kidney Diseases, Hangzhou, China; ^4^ Institute of Nephrology, Zhejiang University, Hangzhou, China; ^5^ Zhejiang Clinical Research Center of Kidney and Urinary System Disease, Hangzhou, China

**Keywords:** minimal change disease, serum IgE level, remission, relapse, risk factor

## Abstract

**Background:**

Previous reports showed that some patients with minimal change disease (MCD) had high serum immunoglobulin E (IgE) levels. This study aimed to explore the proportion of MCD patients with high serum IgE levels and evaluate the correlation between serum IgE levels and MCD remission and relapse.

**Methods:**

This study enrolled 222 new-onset patients with renal biopsy-confirmed MCD from October 2012 to October 2019 at the First Affiliated Hospital of Zhejiang University in Hangzhou, China. Patients’ demographics and clinical parameters were analyzed.

**Results:**

The results indicated that 70.3% of 222 MCD patients had high serum IgE levels (IgE > 100.0 IU/mL). Moreover, 134 patients were treated with glucocorticoids alone and divided into the low- and high-IgE groups, according to the median serum IgE level (523.5 IU/mL). The mean time to complete remission of the low- and high-IgE groups was 29.0 ± 2.2 and 45.7 ± 4.2 days, respectively (log-rank test; *P* = 0.002). The mean time to total remission was 19.1 ± 1.4 and 31.6 ± 3.2 days of the low- and high-IgE groups, respectively (log-rank test; *P* < 0.001). The mean time to first relapse in the low- and high-IgE groups was 701.2 ± 65.0 and 425.0 ± 52.6 days, respectively (log-rank test; *P* = 0.002). Serum IgE ≥ 523.5 IU/mL was an independent correlation factor affecting the patients’ remission and relapse.

**Conclusion:**

Serum IgE level was an independent correlation factor for MCD remission and relapse. MCD patients with high serum IgE levels were prone to delayed remissions and early relapses.

## 1 Introduction

Minimal change disease (MCD) is a common pathological type of idiopathic nephrotic syndrome (INS). MCD accounts for 70%–90%, 50%, and 10%–15% of patients with INS in children < 10 years old, children > 10 years old, and adults, respectively (1). Typical MCD clinical manifestations include hypoalbuminemia and massive proteinuria, possibly accompanied by edema and hyperlipidemia (1). Glucocorticoids are usually the first choice for initial immunosuppressive therapy in patients with MCD (2). About 90% and 70% of children and adults with MCD, respectively, can achieve complete remission after receiving a course of glucocorticoid treatment but are prone to relapse (3). About 56%–76% of MCD patients will experience at least one relapse, and some patients may experience frequent relapses or steroid dependence (4). Changing the therapeutic regimen in time is crucial for these patients. Therefore, evaluating the clinical efficacy of glucocorticoids in MCD is important.

Reports in the 1970s showed that the serum immunoglobulin E (IgE) levels of several MCD patients were higher than normal (5). Serum IgE level was low in normal conditions, and elevated level was usually associated with allergic reactions (6). Several case reports reported the onset of nephrotic syndrome caused by food, allergen inhalation, insect bites, and vaccination (7–10). Numerous reports indicated that INS could be precipitated by allergic reactions, and INS patients could exhibit increased serum IgE levels (11). However, many MCD patients with high serum IgE levels had no history of allergy. Therefore, although some INS cases were associated with allergies, evidence that INS was a type of allergic disorder was weak (11). Shu et al. (12) reported that the serum IgE levels in MCD patients with frequent relapses were significantly higher than that in patients with non-relapse or infrequent-relapse, indicating that high serum IgE levels may be related to frequent MCD relapse. But the relevant studies on the correlation between serum IgE levels and prognosis of MCD were lacking. This study aims to explore the proportion of MCD patients with high serum IgE levels and evaluate the correlation between serum IgE levels and MCD remission and relapse.

## 2 Methods

### 2.1 Study Design and Population

This retrospective observational study was performed at a single center, the Kidney Disease Center of the First Affiliated Hospital, College of Medicine, Zhejiang University (Hangzhou, China), from October 2012 to October 2019. The study complied with the Declaration of Helsinki. The Clinical Research Ethics Committee of the First Affiliated Hospital, Zhejiang University School of Medicine, provided ethical approval and waived informed consent.

The study included patients who met the following criteria: (1) 24-h urinary protein ≥ 3.5 g/day or urine protein to creatinine ratio (UP/Cr) ≥ 3.5 g/g for adults, and UP/Cr ≥ 2.0 g/g or ≥ 3+ on urine dipstick for children; (2) serum albumin < 30 g/L; (3) pathologically proven MCD by renal biopsy; and (4) new-onset of disease or discontinuation of immunosuppressive therapy for more than 1 year. Patients were excluded if they had any of the following conditions: (1) infectious diseases (e.g. hepatitis B, AIDS, syphilis, and tuberculosis); (2) malignant tumors; (3) connective tissue diseases; (4) diabetes mellitus; and (5) missing data on serum IgE levels at the onset.

### 2.2 Data Collection

As shown in **Table 1**, patiens’ demographics and clinical parameters at the time of renal biopsy were collected, including gender, age, disease duration, body mass index, serum total IgE level, serum albumin (Alb), serum creatinine (SCr), estimated glomerular filtration rate (eGFR), serum uric acid (UA), serum triglyceride (TG), serum total cholesterol (TC), urine protein to creatinine ratio (UP/Cr), systolic blood pressure, diastolic blood pressure, fasting blood glucose, allergic history, comorbidities, treatment regimen, *etc.*

**Table 1:** Baseline characteristics of patients with minimal change disease.

	Mean ± SD/median (Q1, Q3)/ *n* (%)			*P* ^*^
	Overall, *n* = 222	Low-IgE, *n* = 111	High-IgE, *n* = 111	
IgE level, IU/mL	389.5 (79.5, 1087.2)	79.4 (40.0, 157.0)	1090.0 (685.5, 1807.5)	**<0.001**
Female	88 (39.6%)	64 (57.7%)	24 (21.6%)	**<0.001**
Age, years old	25.5 (19.0, 43.8)	33.0 (20.0, 50.5)	22.0 (18.0, 28.5)	**<0.001**
Adult	182 (82.0%)	96 (86.5%)	86 (77.5%)	0.081
Disease duration, days	10.0 (7.0, 20.0)	10.0 (7.0, 20.0)	10.0 (7.0, 25.5)	0.900
Treatment regimens				**0.013**
GC	134 (60.4%)	58 (52.3%)	76 (68.5%)	
GC + TAC	58 (26.1%)	30 (27.0%)	28 (25.2%)	
TAC	13 (5.9%)	12 (10.8%)	1 (0.9%)	
GC + CsA	7 (3.2%)	3 (2.7%)	4 (3.6%)	
GC + CTX	2 (0.9%)	2 (1.8%)	0 (0.0%)	
GC + RTX	1 (0.5%)	1 (0.9%)	0 (0.0%)	
Non-immunosuppressive treatment	7 (3.2%)	5 (4.5%)	2 (1.8%)	
BMI, kg/m^2^	22.7 (20.8, 25.8)	22.4 (20.8, 24.7)	23.1 (20.8, 26.4)	0.350
Allergy	32 (14.4%)	18 (16.2%)	14 (12.6%)	0.445
FBG, mmol/L	4.4 (4.1, 4.8)	4.4 (4.1, 4.9)	4.3 (4.0, 4.8)	0.252
SBP, mmHg	121.6 ± 13.4	120.4 ± 13.5	122.8 ± 13.3	0.180
DBP, mmHg	75.2 ± 9.9	75.6 ± 9.3	74.8 ± 10.5	0.565
Hypertension	30 (13.5%)	18 (16.2%)	12 (10.8%)	0.239
Alb, g/L	17.6 (15.2, 21.0)	18.3 (15.4, 21.9)	17.2 (14.7, 19.8)	**0.013**
SCr, μmol/L	79.5 (62.0, 102.0)	73.0 (60.5, 94.5)	84.0 (66.5, 117.5)	**0.006**
eGFR, mL/(min × 1.73 m^2^)	96.9 ± 38.0	96.2 ± 37.3	97.6 ± 38.7	0.788
eGFR ≥ 90 mL/(min × 1.73 m^2^)	130 (58.6%)	66 (59.5%)	64 (57.7%)	0.785
eGFR < 90 mL/(min × 1.73 m^2^)	92 (41.4%)	45 (40.5%)	47 (42.3%)
UA, μmol/L	402.3 ± 124.8	374.9 ± 121.7	429.7 ± 122.4	**<0.001**
Abnormal UA level	89 (40.1%)	35 (31.5%)	54 (48.6%)	**0.009**
AKI	39 (17.6%)	14 (12.6%)	25 (22.5%)	0.052
TG, mmol/L	2.0 (1.5, 3.0)	2.0 (1.5, 2.8)	2.2 (1.5, 3.2)	0.623
TG ≤ 1.7 mmol/L	77 (34.7%)	37 (33.3%)	40 (36.0%)	0.672
TG > 1.7mmol/L	145 (65.3%)	74 (66.7%)	71 (64.0%)
TC, mmol/L	9.7 (7.7, 11.9)	8.7 (7.1, 11.3)	10.1 (8.3, 12.3)	**0.010**
TC ≤ 5.86 mmol/L	16 (7.2%)	11 (9.9%)	5 (4.5%)	0.119
TC > 5.86 mmol/L	206 (92.8%)	100 (90.1%)	106 (95.5%)
UP/Cr, g/g	4.9 (3.7, 7.3)	5.1 (3.8, 7.4)	4.7 (3.6, 7.0)	0.323

AKI, acute kidney injury; Alb, albumin; BMI, body mass index; CsA, cyclosporine; CTX, cyclophosphamide; DBP, diastolic blood pressure; eGFR, estimated glomerular filtration rate; FBG, fasting blood glucose; GC, glucocorticoids; Q1, lower quartile; Q3, upper quartile; RTX, rituximab; SBP, systolic blood pressure; SCr, serum creatinine; TAC, tacrolimus; TC, total cholesterol; TG, triglyceride; UA, uric acid; UP/Cr, urine protein to creatinine ratio. ^*^P High-IgE vs. Low-IgE. P < 0.05 was shown in bold.

The eGFR was calculated using the four-variable Modification of Diet in Renal Disease Study equation (13). The serum total IgE levels of MCD patients were analyzed using the ImmunoCAP system (14). The normal value of serum total IgE was ≤ 100 IU/mL.

### 2.3 Immunosuppressive Treatment Regimens

For the new-onset biopsy-proven MCD patients, daily prednisolone (0.5-1.0 mg/kg/d, up to 80 mg/d) was generally used as the initial immunosuppressive treatment, and was maintained for 2-4 weeks if patients achieved complete remission or for a maximum of 16 weeks if patients didn’t achieve complete remission. After remission, the dosage of glucocorticoids was tapered over 6 months. For relapse patients, the same initial dosage of glucocorticoids was used and was gradually tapered after remission was achieved. For patients with a contraindication to or intolerance of high-dose glucocorticoids, and patients with frequent relapses or steroid dependence, second-line agents such as cyclophosphamide, tacrolimus, cyclosporine, or rituximab were used. The choices of second-line agents were up to the individual nephrologists.

### 2.4 Follow-Up

Patients’ clinical parameters at 1, 2, 3, 6, and 12 months were collected and analyzed. And the subsequent follow-ups were also recorded. The data were collected at specific time points if patients achieved remission or relapse at other times (within 1 month or beyond 12 months, for example). The last follow-up point was the latest clinical visit available in the follow-up system. The tolerated time-frame for follow-up time points were 30 days ± 1 week in this study. Clinical parameters included serum IgE levels, Alb, SCr, eGFR, UA, TG, TC, 24-h urine protein, and UP/Cr.

### 2.5 Outcomes Definition

The primary endpoint of this study was remission and relapse. Remission included partial remission (PR), complete remission (CR), and total remission (TR). The secondary endpoint included time to remission and time to first relapse. These indexes were defined as follows (15):

For adults, PR was the decrease in 24-h urine protein to < 3.5 g/day but > 0.3 g/day or in UP/Cr to < 3.5 g/g but > 0.3 g/g with a 50% reduction from its peak value. CR was the serum albumin ≥ 30 g/L with a decrease in 24-h urine protein to ≤ 0.3 g/day or in UP/Cr to ≤ 0.3 g/g. For children, PR was the decrease in UP/Cr to < 2.0 g/g but > 0.2 g/g with a 50% reduction from its peak value. CR was the serum albumin ≥ 30 g/L with a decrease in UP/Cr to ≤ 0.2 g/g or trace or negative results on repeat urine albumin dipstick.

TR was the achievement of PR or CR.

Time to remission was the time interval from treatment initiation to the first day of remission.

For adults, relapse was the increase in 24-h urine protein to ≥ 3.5 g/day or in UP/Cr to ≥ 3.5 g/g in patients who underwent TR with a 50% increase from its valley value. For children, relapse was the increase in urine dipstick to ≥ 3+ or in UP/Cr to ≥ 2.0 g/g in patients who underwent TR with a 50% increase from its valley value.

Time to the first relapse was the time interval from TR initiation to the day when the first relapse occurred (16).

Steroid resistance was defined as not achieving remission despite at least 16 weeks of prednisone (1 mg/kg/d) treatment.

Frequent relapse was defined as 2 or more relapses per 6 months (or 4 or more relapses per 12 months).

Steroid dependence was defined as relapse during corticosteroid therapy or within 2 weeks of discontinuing corticosteroid therapy.

### 2.6 Statistical Analysis

The Kolmogorov–Smirnov test determined whether the continuous variables conform to the normal distribution. Normally distributed continuous variables were represented by mean ± SD, and the comparison between the two groups was performed using the unpaired *t-*test. Non-normally distributed continuous variables were represented by median (lower quartile, upper quartile), and the Mann–Whitney *U* test was used to compare the two groups. Categorical variables were expressed as frequency (percentage), and comparison between groups was performed by chi-square test, continuity-corrected chi-square test, or Fisher’s exact test. A two-sided test was performed on all data, and *P* < 0.05 was regarded as statistically significant.

The remission rate and the probability of the first relapse between the two groups were estimated using the Kaplan–Meier method, and survival curves were compared with the log-rank test. Cox proportional hazards analysis model was used to explore the effects of different variables on MCD remission and relapse. Variables with *P* < 0.1 in the univariate analysis were included in the multivariate analysis with covariates. The independent correlation factors for the endpoint event were obtained using forward stepwise regression (α_included_ = 0.05 and α_excluded_ = 0.10).

The SPSS 24.0, GraphPad Prism 9.0, R 4.0.3, and EmpowerStats software were used for data analysis in this study.

## 3 Results

### 3.1 Demographic and Clinical Features

**Figure 1:**
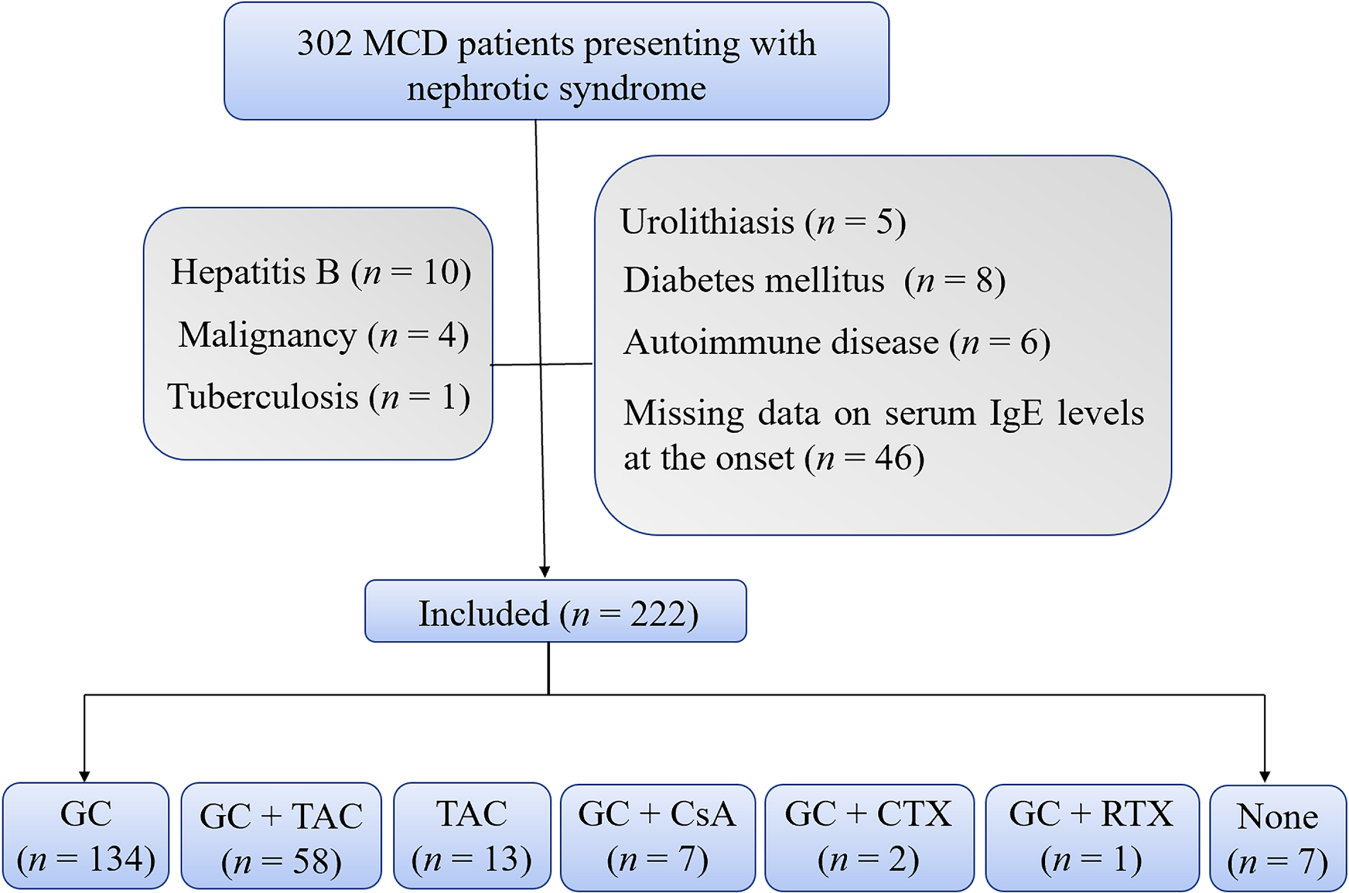
Flowchart. The flowchart shows 222 enrolled patients with 134 patients treated with glucocorticoids alone and 88 patients treated with other treatment regimens. CsA, cyclosporine; CTX, cyclophosphamide; GC, glucocorticoids; TAC, tacrolimus; RTX, rutuximab.

As shown in **Figure 1**, a total of 222 MCD patients were enrolled in this study. The baseline characteristics of these patients were listed in **Table 1**. The median (Q1, Q3) of the age was 25.5 (19.0, 43.8) years old, and the range of the age was 14.0-81.0 years old. A total of 182 patients (82.0%) were adults. The median serum IgE level of these patients was 389.5 (79.5, 1087.2) IU/mL. And 156 (70.3%) patients had high serum IgE levels (IgE > 100.0 IU/mL) at the disease onset. Of the 222 patients, 134 patients received glucocorticoids alone as their initial immunosuppressive treatment, 58 patients received glucocorticoids plus tacrolimus, 13 patients received tacrolimus alone, 7 patients received glucocorticoids plus cyclosporin, 2 patients received glucocorticoids plus cyclophosphamide, 1 patient received glucocorticoids plus rituximab, and 7 patients didn’t receive any immunosuppressive treatment.

And the 134 patients who received glucocorticoids alone were included for further analyses to explore the correlation between serum IgE levels and the efficacy of glucocorticoids for MCD. As shown in **Table 2**, of the included patients, 46 were females which accounted for 34.3% of the total population. 27 were children which accounted for 20.1% of the total population. And the median age of the patients was 24.0 (19.0, 43.8) years old. The median follow-up period of the patients was 15.2 (12.2, 46.6) months. A total of 22 patients that accounted for 16.4% of the total population had allergic history, including bronchial asthma, allergic rhinitis, atopic dermatitis, urticaria, and other allergic conditions. 33 patients that accounted for 24.6% of the total population had infections at the disease onset, including upper respiratory tract infections, suppurative tonsillitis, pneumonia, and gastroenteritis. The median serum IgE level was 523.5 (91.1, 1230.8) IU/mL and was set as the cutoff point, and 134 MCD patients were equally divided into two groups: low-IgE (IgE < 523.5 IU/mL) and high-IgE (IgE ≥ 523.5 IU/mL) groups.

**Table 2:** Baseline characteristics of patients with minimal change disease treated with glucocorticoids.

	Mean ± SD/median (Q1, Q3)/ *n* (%)			*P* ^*^
	Overall, *n* = 134	Low-IgE, *n* = 67	High-IgE, *n* = 67	
IgE level, IU/mL	523.5 (91.1, 1230.8)	90.6 (42.0, 256.5)	1238.0 (808.5, 2239.5)	**<0.001**
Female	46 (34.3%)	33 (49.3%)	13 (19.4%)	**<0.001**
Age, years old	24.0 (19.0, 43.8)	32.0 (19.0, 50.0)	21.0 (18.0, 28.0)	**0.024**
Adult	107 (79.9%)	55 (82.1%)	52 (77.6%)	0.518
Disease duration, days	10.0 (7.0, 20.8)	10.0 (7.0, 20.0)	10.0 (7.0, 25.5)	0.881
Follow-up period, months	15.2 (12.2, 46.6)	15.7 (12.2, 49.5)	14.7 (12.2, 42.2)	0.805
BMI, kg/m^2^	23.3 (20.9, 25.8)	23.6 (21.4, 25.9)	22.5 (20.8, 25.5)	0.259
Alcohol	19 (14.2%)	9 (13.4%)	10 (14.9%)	0.804
Smoking	28 (20.9%)	10 (14.9%)	18 (26.9%)	0.089
Infections	33 (24.6%)	14 (20.9%)	19 (28.4%)	0.316
Upper respiratory tract infections	24 (17.9%)	10 (14.9%)	14 (20.9%)	0.367
Suppurative tonsillitis	2 (1.5%)	1 (1.5%)	1 (1.5%)	1.000
Pneumonia	4 (3.0%)	2 (3.0%)	2 (3.0%)	1.000
Gastroenteritis	3 (2.2%)	1 (1.5%)	2 (3.0%)	1.000
Allergy	22 (16.4%)	10 (14.9%)	12 (17.9%)	0.641
Bronchial asthma	2 (1.5%)	2 (3.0%)	0 (0.0%)	0.496
Allergic rhinitis	4 (3.0%)	1 (1.5%)	3 (4.5%)	0.619
Atopic dermatitis	6 (4.5%)	2 (3.0%)	4 (6.0%)	0.680
Urticaria	3 (2.2%)	2 (3.0%)	1 (1.5%)	1.000
Other allergic conditions	9 (6.7%)	4 (6.0%)	5 (7.5%)	1.000
FBG, mmol/L	4.4 (4.1, 4.8)	4.5 (4.2, 4.8)	4.3 (4.0, 4.7)	0.059
SBP, mmHg	122.2 ± 12.2	122.3 ± 13.0	122.1 ± 11.5	0.922
DBP, mmHg	75.0 ± 9.6	76.2 ± 9.9	73.7 ± 9.2	0.133
Hypertension	20 (14.9%)	14 (20.9%)	6 (9.0%)	0.052
Alb, g/L	17.6 (15.3, 20.8)	18.3 (15.7, 21.4)	17.0 (14.6, 19.8)	0.055
SCr, μmol/L	82.5 (62.0, 107.2)	75.0 (61.0, 102.5)	84.0 (65.0, 115.0)	0.295
eGFR, mL/(min × 1.73 m^2^)	97.1 ± 41.6	94.0 ± 41.5	100.1 ± 41.8	0.402
eGFR ≥ 90 mL/(min × 1.73 m^2^)	77 (57.5%)	38 (56.7%)	39 (58.2%)	0.861
eGFR < 90 mL/(min × 1.73 m^2^)	57 (42.5%)	29 (43.3%)	28 (41.8%)
Eosinophilia	12 (9.0%)	1 (1.5%)	11 (16.4%)	**0.006**
UA, μmol/L	417.4 ± 129.5	401.6 ± 131.5	433.2 ± 126.6	0.159
Abnormal UA level	67 (50.0%)	32 (47.8%)	35 (52.2%)	0.604
AKI	36 (26.9%)	16 (23.9%)	20 (29.9%)	0.436
TG, mmol/L	2.0 (1.5, 3.0)	2.2 (1.6, 2.9)	1.9 (1.4, 3.2)	0.245
TG ≤ 1.7 mmol/L	51 (38.1%)	20 (29.9%)	31 (46.3%)	0.050
TG > 1.7 mmol/L	83 (61.9%)	47 (70.1%)	36 (53.7%)
TC, mmol/L	9.4 (7.5, 12.0)	8.8 (7.1, 11.7)	9.7 (8.2, 12.2)	0.186
TC ≤ 5.86 mmol/L	10 (7.5%)	6 (9.0%)	4 (6.0%)	0.742
TC > 5.86 mmol/L	124 (92.5%)	61 (91.0%)	63 (94.0%)
UP/Cr, g/g	5.0 (3.7, 7.1)	4.8 (3.7, 7.2)	5.0 (4.2, 6.9)	0.772
Total steroid dosages, g	1.2 (0.8, 2.2)	1.0 (0.7, 1.8)	1.4 (0.9, 2.9)	**0.008**
Steroid-resistance	2 (1.5%)	0 (0.0%)	2 (3.0%)	0.496
Steroid-dependence	30 (22.4%)	10 (14.9%)	20 (29.9%)	0.038
Frequent relapse	9 (6.7%)	1 (1.5%)	8 (11.9%)	0.033

AKI, acute kidney injury; Alb, albumin; BMI, body mass index; DBP, diastolic blood pressure; eGFR, estimated glomerular filtration rate; EOS, Eosinophil; FBG, fasting blood glucose; GC, glucocorticoids; Q1, lower quartile; Q3, upper quartile; SBP, systolic blood pressure; SCr, serum creatinine; TC, total cholesterol; TG, triglyceride; UA, uric acid; and UP/Cr, urine protein to creatinine ratio. ^*^P High-IgE vs. Low-IgE. P < 0.05 was shown in bold.

The median serum IgE level was 90.6 (42.0, 256.5) and 1238.0 (808.5, 2239.5) IU/mL in the low- and high-IgE groups, respectively. In the high-IgE group, the ages of the patients were significantly lower than that in the low-IgE group [21.0 (18.0, 28.0) *vs.* 32.0 (19.0, 50.0); *P* = 0.024]; the proportion of female patients was significantly lower than that in the low-IgE group (19.4% *vs.* 49.3%, *P* < 0.001); and the proportion of patients with eosinophilia was significantly higher than that of the low-IgE group (16.4.% *vs.* 1.5%; *P* = 0.006). Moreover, the total dosages of glucocorticoids used in patients in the high-IgE group were significantly higher than that in the low-IgE group [1.4 (0.9, 2.9) *vs.* 1.0 (0.7, 1.8); *P* = 0.008]. There were no significant differences in the allergic history or other baseline parameters between the two groups (*P* > 0.05).

### 3.2 Outcomes

#### 3.2.1 Remission

The Kaplan–Meier curves were used for analysis to compare the cumulative remission rate of MCD patients in the low- and the high-IgE groups. And the Cox regression model was used to further explore the correlation between serum IgE levels and the remission rate of patients treated with glucocorticoids.

**Figure 2:**
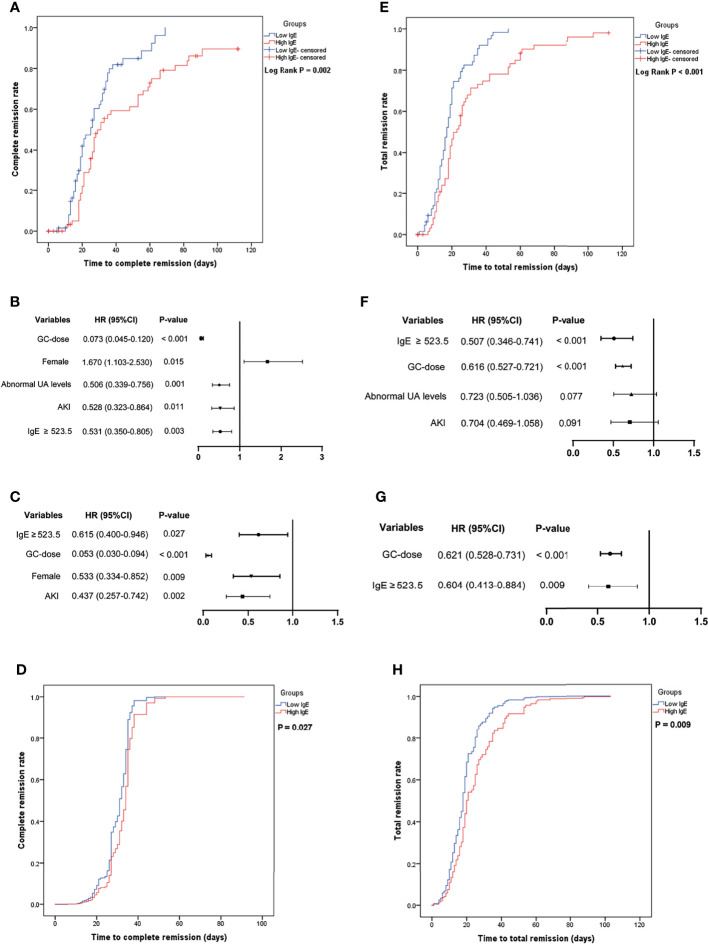
The cumulative remission rate of minimal change disease (MCD) in the low- and high-IgE groups and the identification of independent correlation factors for remission. **(A)** Cumulative complete remission rate. Independent correlation factors for complete remission by univariate **(B)** and multivariate **(C)** cox regression analysis. **(D)** Serum IgE levels were independent correlation factors for complete remission of MCD. **(E)** Cumulative total remission rate. Independent correlation factors for total remission by univariate **(F)** and multivariate **(G)** cox regression analysis. **(H)** Serum IgE levels were independent correlation factors for total remission of MCD. *AKI*, acute kidney injury; *GC-dose*, the dosages of glucocorticoids; *MCD*, minimal change disease, and *UA*, uric acid.

**Figure 2A** shows that the average time to CR was 29.0 ± 2.2 and 45.7 ± 4.2 days in the low- and high-IgE groups (log-rank test; *P* = 0.002), respectively. **Figures 2B, C** shows the independent correlation factors for CR of MCD. Serum IgE ≥ 523.5 IU/mL (hazard ratio [HR] = 0.615, 95% confidence interval [CI] = 0.400–0.946; *P* =0.027), acute kidney injury (AKI; HR = 0.437, 95% CI = 0.257–0.742; *P* = 0.002), dosages of glucocorticoids (GC-dose; HR = 0.053, 95% CI = 0.030–0.094; *P* < 0.001), and female (HR = 0.533, 95% CI = 0.334–0.852; *P* = 0.009) were independent CR correlation factors. **Figure 2D** shows the cumulative CR rates of MCD patients in the low- and the high-IgE groups after adjusting for AKI, UA levels, age, eGFR, GC-dose, and gender in the multivariate Cox regression model. The cumulative CR rate of MCD patients in the high-IgE group was significantly lower than that in the low-IgE group (*P* = 0.027).

**Figure 2E** shows that the average time to TR was 19.1 ± 1.4 and 31.6 ± 3.2 days in the low- and high-IgE groups (log-rank test; *P* < 0.001), respectively. **Figures 2F, G** shows the independent correlation factors for TR of MCD. Serum IgE ≥ 523.5 IU/mL (HR = 0.604, 95% CI = 0.413–0.884; *P* =0.009), and GC-dose (HR = 0.621, 95% CI = 0.528–0.731; *P* < 0.001) were independent TR correlation factors. As shown in **Figure 2H**, after adjusting for age, AKI, GC-dose, and UA levels in the multivariate Cox regression model, the cumulative TR rate of MCD patients in the high-IgE group was significantly lower than that in the low-IgE group (*P* = 0.009).

Moreover, 2 patients exhibited steroid-resistance in the high-IgE group (**Table 2**). One of them (IgE = 2874 IU/mL) was re-diagnosed with focal segmental glomerular sclerosis (FSGS) after repeat renal biopsy 5 months later. The other one (IgE = 904 IU/mL) achieved complete remission after a combined treatment of tacrolimus plus glucocorticoids for 5 months, but experienced frequent relapses later during the treatment period, and was suspected as FSGS clinically. In addition, 10 (14.9%) and 20 (29.9%) patients were steroid-dependent in the low- and high-IgE groups, respectively (*P* =0.038). And 8 (11.9%) patients in the high-IgE group and 1 (1.5%) patient (IgE = 420 IU/mL) in the low-IgE group experienced frequent relapses (*P* =0.033).

#### 3.2.2 Relapse

**Figure 3:**
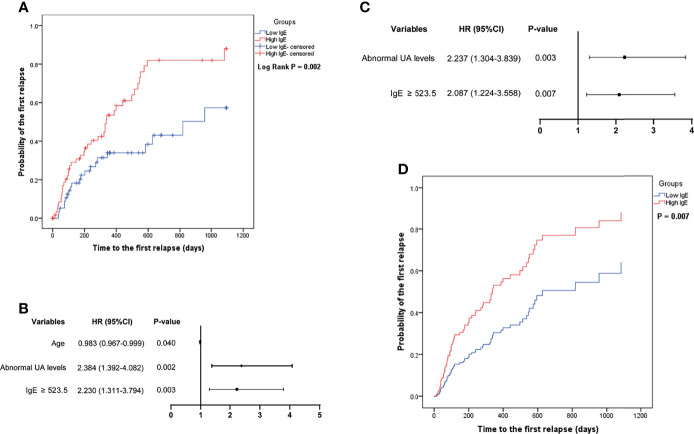
Comparison of the probability of the first relapse of minimal change disease in the low- and high-IgE groups **(A)**, and the identification of independent correlation factors for relapse by univariate **(B)** and multivariate **(C)** cox regression analysis. **(D)** Serum IgE levels were independent correlation factors for relapse of minimal change disease. *UA*, uric acid.

**Figure 3A** shows that the mean time to the first relapse in the low- and high-IgE groups was 701.2 ± 65.0 and 425.0 ± 52.6 days, respectively (log-rank test; *P* = 0.002). **Figures 3B, C** shows that serum IgE ≥ 523.5 IU/mL (HR = 2.087, 95% CI =1.224–3.558; *P* = 0.007), and abnormal UA level (HR = 2.237, 95% CI = 1.304–3.839; *P* = 0.003) were independent risk factors for the first relapse in MCD patients. In the multivariate Cox regression model, the probability of the first relapse of MCD patients (**Figure 3D**) in the high-IgE group was significantly higher than that in the low-IgE group (*P* = 0.007) after adjusting for age and UA levels.

#### 3.2.3 Laboratory Data

**Figure 4:**
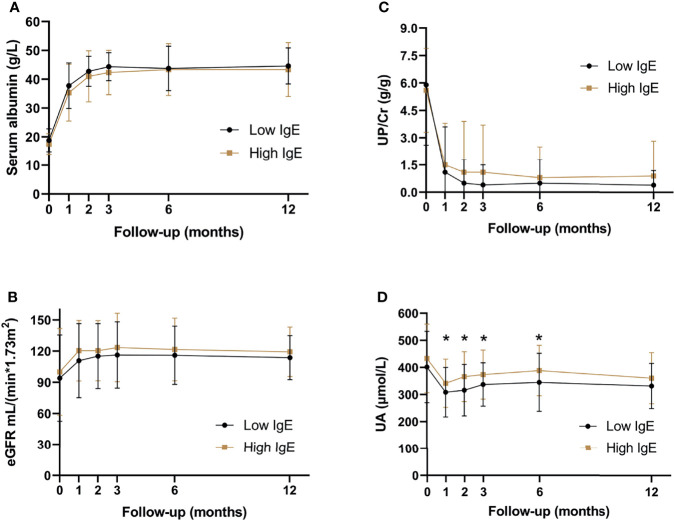
Serum biochemical indexes of patients with minimal change disease during follow-up period. **(A)** serum albumin levels, **(B)** estimated glomerular filtration rate, **(C)** urine protein-creatinine ratio, and **(D)** serum uric acid levels. *P < 0.05, significant difference between the high- and low-IgE groups.

Serum albumin, eGFR, UP/Cr, and UA levels were compared between the two groups during the follow-up period. **Figure 4** shows that the UA levels in the high-IgE group were significantly higher than that in the low-IgE group at month 1, 2, 3, and 6 of follow-up (*P* < 0.05), and there were no significant differences between serum albumin, eGFR, and UP/Cr of the MCD patients in the two groups at month 0, 1, 2, 3, 6, and 12 of follow-up (*P* > 0.05). Although 26.9% of the patients experienced AKI at the onset (as shown in **Table 2**), their renal functions gradually recovered as the proteinuria disappeared.

## 4 Discussions

70.3% of the 222 MCD patients had high serum IgE levels at the onset in this study, including 75.0% of children and 69.2% of adults (*P* = 0.470). This result was consistent with previous reports. A previous study including 46 Chinese adult MCD patients found that 83.7% of the patients had high serum IgE levels, although only one patient had allergic history (17). Another study included 32 children with MCD and reported that 62.5% of the patients had high serum IgE levels (18). Elevated IgE levels usually indicated the occurrence of allergy (19). Though there were no significant differences in the history of allergy between the low- and high-IgE groups in this study, a higher percentage of eosinophilia was observed in the high-IgE group, indicating that the allergic condition may exist. Ni et al. (20) and Cheung et al. (21) reported that serum IgE levels of MCD patients were higher in the atopic subgroup than that in the non-atopic subgroup at the time of remission. We assessed the IgE levels when patients were in remission. However, only 61 of the 134 patients had data on serum IgE levels at the time of disease remission, including 12 patients in the atopic subgroup and 49 in the non-atopic subgroup. When we compared IgE levels between non-atopic *vs.* atopic subgroups at the time of remission, there was no significant difference between the two groups [145.0 (41.3–435.0) *vs.* 166.0 (76.3–501.5); *P* = 0.599]. Because there were many missing data on IgE levels at the time of remission, which was a limitation of retrospective studies, we did not include these data in the results. Further analysis of IgE levels at the time of MCD remission will be required in the prospective studies.

It’s known that children and adults differed in the prognosis of MCD. In this cohort, there were 40 children patients with the ages ranging from 14.0-17.0 years old, which accounted for 18.0% of the 222 patients. And the age was recognized as a confounding factor and was included in our analysis. There was no difference in the proportion of children between the low- and high-IgE groups (17.9% *vs.* 22.4%, *P* = 0.518). Results showed that age was not an independent correlation factor for remission or relapse. Serum IgE levels were independent correlation factors for remission and relapse after adjusting for age and other covariates in the multivariate cox regression model.

In this cohort, 134 patients received glucocorticoids alone, and the remaining 88 patients received other regimens, of which 58 patients received glucocorticoids plus tacrolimus. However, the time of adding tacrolimus to these 58 patients was different due to the shortcomings of retrospective studies, resulting in inconsistent treatment regimens and difficulty in further comparative analysis. And the number of patients in other treatment groups were insufficient for comparative analysis. Therefore, these patients were not included for further analysis. And the 134 patients treated with glucocorticoids alone were included in the further analysis.

Previous studies indicated that the serum IgE levels might serve as a prognostic indicator for steroid responsiveness in MCD patients (12, 22). A 2015 study including 30 children with steroid-sensitive nephrotic syndrome reported that patients with normal IgE levels mostly responded in week 1 after steroid therapy, and patients with high serum IgE levels mostly responded to glucocorticoids in weeks 2 or 3 after therapy (23). Another study compared the clinical characteristics of INS patients with normal IgE and high IgE levels, and reported that the high-IgE group required a significantly longer time to remission, and was more susceptible to frequent relapse (18). In this study, a delayed remission and an early relapse for MCD patients in the high-IgE group was observed, indicating that the serum IgE levels were closely related to glucocorticoid responsiveness in MCD patients, which was consistent with previous studies.

Up to 85% of MCD patients will relapse within 5 years, although glucocorticoids are effective in treating most patients with first-onset MCD (24). In this study, the time to first relapse of MCD patients found in the high-IgE group was significantly shorter than that in the low-IgE group, consistent with other studies’ results (25–27).And a higher percentage of MCD patients were steroid-dependent in the high-IgE group than that in the low-IgE group in this study, and more patients experienced frequent relapses in the high-IgE group, as well. A previous study reported that the mean serum IgE levels of pediatric MCD patients at the time of relapse in frequent-relapse group was more than 3 times higher than that in infrequent-relapse group (25). And the serum IgE level decreased to normal at the time of remission in infrequent-relapse group, but it was still high in frequent-relapse group, indicating a persistent immune disorder in the patients with high IgE levels (25).

The IgE synthesis requires two signals: the first signal is transmitted by the cytokine interleukin (IL)-4 or IL-13 produced by type 2 helper T cells (Th2), and the second signal is transmitted by CD40 and CD40L activation (28). This indicates that the increase in serum IgE levels might be related to Th2 activation (29, 30). Some evidence suggests that the Th2 cytokine, IL-13, may play a potential regulatory role in MCD pathogenesis and high serum IgE levels: multiple reports have shown that serum IL-13 levels in MCD patients are elevated; IL-13 can regulate IgE production; and IL-13 can induce glomerular podocyte damage in animal models and caused MCD-like pathological changes (11, 20, 21). Therefore, IL-13 may drive the onset of the nephrotic syndrome and the increase of serum IgE levels. Thus, IgE is expected to play a role in MCD diagnosis and prognosis evaluation.

## 5 Conclusions

This study investigated the correlation between serum IgE levels and the clinical efficacy of glucocorticoids in MCD. It demonstrated that serum IgE level was an independent correlation factor for MCD remission and relapse. MCD patients with high serum IgE levels were prone to delayed remissions and early relapses. These findings could lay the foundation for further studies on MCD pathogenesis and theranostics.

## Data Availability Statement

The raw data supporting the conclusions of this article will be made available by the authors, without undue reservation.

## Ethics Statement

The studies involving human participants were reviewed and approved by The Clinical Research Ethics Committee of the First Affiliated Hospital, Zhejiang University School of Medicine. The ethics committee waived the requirement of written informed consent for participation

## Author Contributions

HL and LW contributed to the study design, data acquisition, statistical analysis, and manuscript writing. XL, WC, and YZ contributed to data analysis. JC contributed to commentary and revision of the manuscript. All authors contributed to the article and approved the submitted version.

## Conflict of Interest

The authors declare that the research was conducted in the absence of any commercial or financial relationships that could be construed as a potential conflict of interest.

## Publisher’s Note

All claims expressed in this article are solely those of the authors and do not necessarily represent those of their affiliated organizations, or those of the publisher, the editors and the reviewers. Any product that may be evaluated in this article, or claim that may be made by its manufacturer, is not guaranteed or endorsed by the publisher.

## References

[1] Vivarelli M Massella L Ruggiero B Emma F. Minimal Change Disease. Clin J Am Soc Nephro (2017) 12(2):332. doi: 10.2215/CJN.05000516 PMC5293332 27940460

[2] Beck L Bomback AS Choi MJ Holzman LB Langford C Mariani LH. KDOQI US Commentary on the 2012 KDIGO Clinical Practice Guideline for Glomerulonephritis. Am J Kidney Dis (2013) 62(3):403–41. doi: 10.1053/j.ajkd.2013.06.002 23871408

[3] van den Berg JG Weening JJ. Role of the Immune System in the Pathogenesis of Idiopathic Nephrotic Syndrome. Clin Sci (Lond) (2004) 107(2):125–36. doi: 10.1042/cs20040095 15157184

[4] Hogan J Radhakrishnan J. The Treatment of Minimal Change Disease in Adults. J Am Soc Nephrol (2013) 24(5):702–11. doi: 10.1681/asn.2012070734 23431071

[5] Schulte-Wissermann H Gortz W Straub E. IgE in Patients With Glomerulonephritis and Minimal-Change Nephrotic Syndrome. Eur J Pediatr (1979) 131(2):105–11. 10.1007/BF00447472 378666

[6] He JS Narayanan S Subramaniam S Ho WQ Lafaille JJ Curotto de Lafaille MA. Biology of IgE Production: IgE Cell Differentiation and the Memory of IgE Responses. Curr Top Microbiol Immunol (2015) 388:1–19. doi: 10.1007/978-3-319-13725-4_1 25553792

[7] Wittig HJ Goldman AS. Nephrotic Syndrome Associated With Inhaled Allergens. Lancet (1970 7646) 1:542–3. doi: 10.1016/s0140-6736(70)90770-1 4190353

[8] Florido JF Díaz Pena JM Belchi J Estrada JL García Ara MC Ojeda JA. Nephrotic Syndrome and Respiratory Allergy in Childhood. J Investig Allergol Clin Immunol (1992) 2(3):136–40. 1364167

[9] Genova R Sanfilippo M Rossi ME Vierucci A. Food Allergy in Steroid-Resistant Nephrotic Syndrome. Lancet (1987) 1:1315–6. doi: 10.1016/s0140-6736(87)90567-8 2884433

[10] Tareyeva IE Nikolaev AJ Janushkevitch TN. Nephrotic Syndrome Induced by Insect Sting. Lancet (1982) 2:825. doi: 10.1016/s0140-6736(82)92718-0 6126699

[11] Abdel-Hafez M Shimada M Lee PY Johnson RJ Garin EH. Idiopathic Nephrotic Syndrome and Atopy: Is There a Common Link? Am J Kidney Dis (2009) 54(5):945–53. doi: 10.1053/j.ajkd.2009.03.019 PMC2895907 19556042

[12] Shu KH Lian JD Yang YF Lu YS Wang JY. Serum IgE in Primary Glomerular Diseases and Its Clinical Significance. Nephron (1988) 49(1):24–8. doi: 10.1159/000184981 3380216

[13] Levey AS Bosch JP Lewis JB Greene T Rogers N Roth D. A More Accurate Method to Estimate Glomerular Filtration Rate From Serum Creatinine: A New Prediction Equation. Modification of Diet in Renal Disease Study Group. Ann Intern Med (1999) 130(6):461–70. doi: 10.7326/0003-4819-130-6-199903160-00002 10075613

[14] Ewan PW Coote D. Evaluation of a Capsulated Hydrophilic Carrier Polymer (the ImmunoCAP) for Measurement of Specific IgE Antibodies. Allergy (1990) 45(1):22–9. doi: 10.1111/j.1398-9995.1990.tb01080.x 2309986

[15] Rovin BH Adler SG Barratt J Bridoux F Burdge KA Chan TM. KDIGO 2021 Clinical Practice Guideline for the Management of Glomerular Diseases. Kidney Int 100(4s):S1–s276. doi: 10.1016/j.kint.2021.05.021 34556256

[16] Li X Liu Z Wang L Wang R Ding G Shi W. Tacrolimus Monotherapy After Intravenous Methylprednisolone in Adults With Minimal Change Nephrotic Syndrome. J Am Soc Nephrol (2017) 28(4):1286–95. doi: 10.1681/asn.2016030342 PMC5373446 27807213

[17] Huang JJ Hsu SC Chen FF Sung JM Tseng CC Wang MC. Adult-Onset Minimal Change Disease Among Taiwanese: Clinical Features, Therapeutic Response, and Prognosis. Am J Nephrol (2001) 21(1):28–34. doi: 10.1159/000046215 11275629

[18] Youn YS Lim HH Lee JH. The Clinical Characteristics of Steroid Responsive Nephrotic Syndrome of Children According to the Serum Immunoglobulin E Levels and Cytokines. Yonsei Med J (2012) 53(4):715–22. doi: 10.3349/ymj.2012.53.4.715 PMC3381495 22665336

[19] Hamilton RG Hemmer W Nopp A Kleine-Tebbe J. Advances in IgE Testing for Diagnosis of Allergic Disease. J Allergy Clin Immunol Pract (2020) 8(8):2495–504. doi: 10.1016/j.jaip.2020.07.021 32717438

[20] Ni FF Liu GL Jia SL Chen RR Liu LB Li CR. Function of miR-24 and miR-27 in Pediatric Patients With Idiopathic Nephrotic Syndrome. Front Pediatr (2021) 9:651544. doi: 10.3389/fped.2021.651544 33968853 PMC8096900

[21] Cheung W Wei CL Seah CC Jordan SC Yap HK. Atopy, Serum IgE, and Interleukin-13 in Steroid-Responsive Nephrotic Syndrome. Pediatr Nephrol (2004) 19(6):627–32. doi: 10.1007/s00467-004-1438-8 15064938

[22] Groshong T Mendelson L Mendoza S Bazaral M Hamburger R Tune B. Serum IgE in Patients With Minimal-Change Nephrotic Syndrome. J Pediatr (1973) 83(5):767–71. doi: 10.1016/s0022-3476(73)80367-1 4742569

[23] Yilmaz D Yenigun A Sonmez F Kurt Omurlu I. Evaluation of Children With Steroid-Sensitive Nephrotic Syndrome in Terms of Allergies. Ren Fail (2015) 37(3):387–91. doi: 10.3109/0886022x.2014.996087 25598239

[24] Müller-Deile J Schenk H Schiffer M. Minimal Change Disease and Focal Segmental Glomerulosclerosis. Internist (Berl) (2019) 60(5):450–7. doi: 10.1007/s00108-019-0590-y 30887070

[25] Jahan I Hanif M Ali MA Waliullah SM Mia AH. Relationship Between Serum IgE and Frequent Relapse Idiopathic Nephrotic Syndrome. Mymensingh Med J (2011) 20(3):484–9. 21804516

[26] Lee H Yoo KD Oh YK Kim DK Oh KH Joo KW. Predictors of Relapse in Adult-Onset Nephrotic Minimal Change Disease. Med (Baltimore) (2016) 95(12):e3179. doi: 10.1097/md.0000000000003179 PMC4998403 27015208

[27] Tan Y Yang D Fan J Chen Y. Elevated Levels of Immunoglobulin E may Indicate Steroid Resistance or Relapse in Adult Primary Nephrotic Syndrome, Especially in Minimal Change Nephrotic Syndrome. J Int Med Res (2011) 39(6):2307–13. doi: 10.1177/147323001103900629 22289548

[28] Bacharier LB Geha RS. Molecular Mechanisms of IgE Regulation. J Allergy Clin Immunol (2000) 105(2 Pt 2):S547–558. doi: 10.1016/s0091-6749(00)90059-9 10669540

[29] Le Berre L Hervé C Buzelin F Usal C Soulillou JP Dantal J. Renal Macrophage Activation and Th2 Polarization Precedes the Development of Nephrotic Syndrome in Buffalo/Mna Rats. Kidney Int (2005) 68(5):2079–90. doi: 10.1111/j.1523-1755.2005.00664.x 16221207

[30] Sahali D Sendeyo K Mangier M Audard V Zhang SY Lang P. Immunopathogenesis of Idiopathic Nephrotic Syndrome With Relapse. Semin Immunopathol (2014) 36(4):421–9. doi: 10.1007/s00281-013-0415-3 PMC5385209 24402710

